# Supplementary material for: Salivary Cystatin-L2-like of Varroa destructor Causes Lower Metabolism Activity and Abnormal Development in Apis mellifera Pupae
Source: Animals (Basel). 2023 Nov 27;13(23):3660. doi: 10.3390/ani13233660 (PMC10705198; doi:10.3390/ani13233660)
Supplement: Supplementary file 1 [file animals-13-03660-s001.zip › File S1 The optimized DNA sequence and amino acid sequence of Cystatin.pdf]

Supplementary Materials

File S1. The optimized DNA sequence and amino acid sequence of Cystatin

Optimized DNA Sequence

ATGAGTGCAATTTGTTTCGATGTGAGTGCAAGCAATCGTATTGATGCCAATAAGCAGCGTCTGCTGCATCATCGTAA
TCCGGAAGGTCGTAGCAAAAATCCGCGCCGCACCCGTGGTGAATTTCGTCAGCTGGATGCAAAAGAAATTGAAG
AAGAAGAACTGGGCGAACTGGCCCAGTTTGCCGCAACCACCGTGGCCAAAGCCGAAAAACGTGCATTTCATGAT
GCAGTGCTGAATATTACCGAAGCACATCAGCAGACCGTTGCAGGCTGGAATACCAAAATGAAACTGGTTGTTAGT
GAAACCAGTTGCCCGATTCATAATCCGTTTGATGTGGGTCAGTGCATTCCGAAAAATGCAAGTATTACCCGCCAGT
GTGATGTGCTGGTGTATGAACGCCCGTGGGAACATATTAAGGAACTGACCAGCTTTTATTGTCAT

Optimized Protein Sequence

MSAICFDVSASNRIDANKQRLLHHRNPEGRSKNPRRTRGEFRQLDAKEIEEEELGELAQFAATTVAKAEKRAFHDAVL
NITEAHQQTVAGWNTKMKLVVSETSCPIHNPFDVGQCIPKNASITRQCDVLVYERPWEHIKELTSFYCH
