# Supplementary material for: Salivary Cystatin-L2-like of Varroa destructor Causes Lower Metabolism Activity and Abnormal Development in Apis mellifera Pupae
Source: Animals (Basel). 2023 Nov 27;13(23):3660. doi: 10.3390/ani13233660 (PMC10705198; doi:10.3390/ani13233660)

Original Images for Blots/Gels

1. The follow original image of SDS-PAGE is corresponding to the figure 2B in the main article, the right part of the original image with the lane 1-6 labeled was just the figure 2B in the main article. The rest part of the original image (the left six lanes unlabeled) was unrelated to the present work.

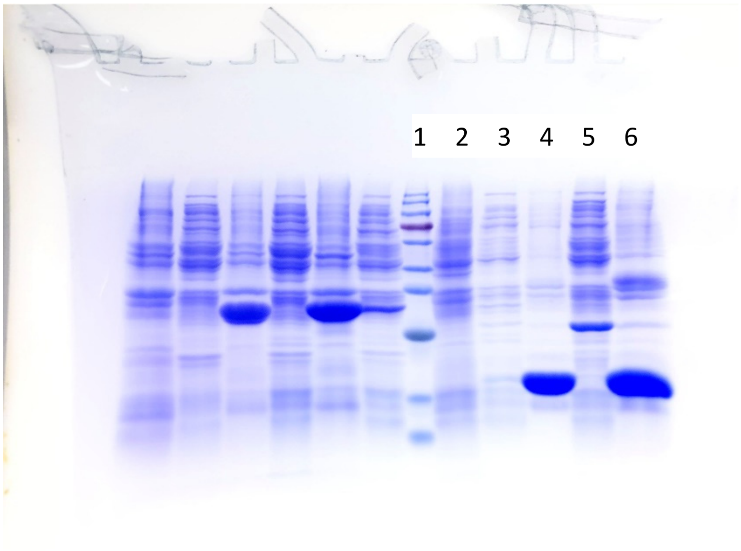

2. The figure 2C in the main article was from the follow original image of Western Blot. The right two lanes labeled was just the figure 2C in the main article. The other part of the Western Blot image was unrelated with the present work and negligible.

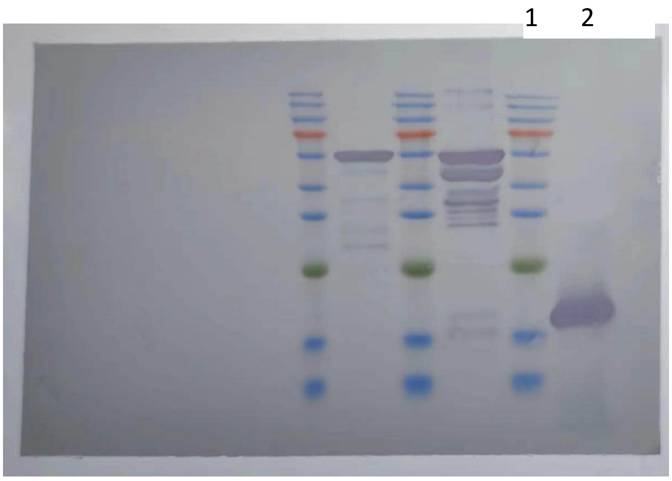

3. The figure 2D in the main article was from the follow original image of SDS-PAGE. The right two lanes labeled was just the figure 2D in the main article. The other part of the SDS-PAGE image was unrelated with the present work and negligible.

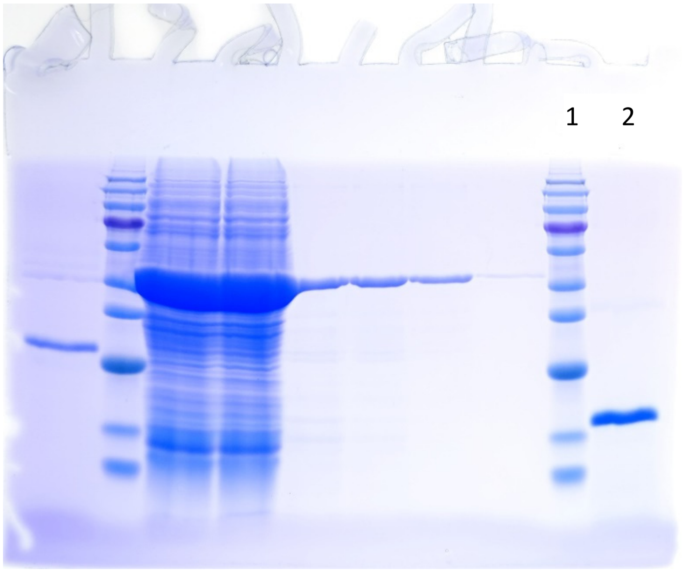

Supplement: Supplementary file 1 [file animals-13-03660-s001.zip › File S2 Original Images for Blots and Gels.pdf]
